# Case Report: Endovascular Treatment of a Giant Distal PICA Aneurysm in Association With a Cerebellar AVM: A Report on Treatment Considerations and a Literature Review

**DOI:** 10.3389/fneur.2020.611377

**Published:** 2020-12-18

**Authors:** Hassan A. Khayat, Christine M. Hawkes, Almunder R. Algird

**Affiliations:** Division of Neurosurgery, Department of Surgery, Hamilton Health Sciences, McMaster University, Hamilton, ON, Canada

**Keywords:** Arteriovenous malformation, prenidal aneurysm, posterior inferior cerebellar artery (PICA), coiling, parent vessel occlusion

## Abstract

**Background:** Distal posterior inferior cerebellar artery (PICA) aneurysms are uncommon intracranial vascular lesions. The coincidence of these aneurysms and Arteriovenous malformation (AVM) is even more rare. Since 1956, a total of 57 cases of distal PICA aneurysms associated with AVM have been reported with clear and adequate description. None of these reports describe a giant prenidal aneurysm at this particular location. The paucity of natural history data as well as lack of consensus about treatment strategies in such cases present a significant challenge that requires an individualized management approach.

**Case Description:** A 68-year-old male presented with recurrent episodes of nausea and vomiting precipitated by physical exertion and change of head position. An MRI of the brain demonstrated a giant partially thrombosed right posterior inferior cerebellar artery (PICA) aneurysm with mass effect on the floor of the fourth ventricle. A conventional cerebral angiogram revealed a giant (3.1 x 3.1 x 2.8cm) distal right PICA pre-nidal aneurysm with two smaller distal PICA aneurysms. An AVM (Spetzler-Martin Grade 1) supplied by the right PICA as well as the right superior cerebellar artery (SCA) was also identified on cerebral angiography (not seen on an MRI). Endovascular coil embolization with parent vessel sacrifice was performed to occlude the giant aneurysm. Due to the asymptomatic nature, low risk of rupture, and the patient's age, AVM treatment was deferred.

**Conclusion:** This paper presents the first case of a giant PICA aneurysm associated with cerebellar AVM. For PICA aneurysm-AVM complexes, meticulous evaluation of the morphology, associated anatomy, and comparative risk analysis for both lesions are key for treatment planning. Distal PICA aneurysms can be treated safely with parent vessel occlusion, particularly in the case of prenidal aneurysms.

## Highlights

- Prenidal distal PICA aneurysms are rare.
- Both the AVM and the aneurysm require thorough and careful assessment.
- Determining the risk of rupture associated with each lesion is important for making treatment decisions.
- Parent artery occlusion is an option for dissecting pseudoaneurysms located distally along the PICA.

## Introduction

Posterior inferior cerebellar artery (PICA) aneurysms are uncommon vascular lesions accounting only for 0.5–3% of all intracranial aneurysms (1). Most of these aneurysms arise at the proximal segment of PICA. However, while distal aneurysms are rare, they are most often prenidal with a more distally located Arteriovenous malformation (AVM) (2). The explanation of how these lesions might coincide is yet to be elucidated. The current understanding suggests a hemodynamic stress and/or a congenital defect as an underlying mechanism (3, 4). The combination of a distal PICA aneurysm and an AVM has been sporadically described in the literature over the past 70 years; however, there have been no reports associating a giant symptomatic PICA aneurysm with a small asymptomatic AVM. In 1956, Patterson et al. first reported the coincidence of a distal PICA aneurysm and an associated cerebellar AVM (3). In 1999, the number of cases reported increased to 27 (4). A recent report in 2017 counted a total of 51 cases in the literature having this combination with five cases identified at their institution (5). Since this combination is rarely encountered, case reports such as this contribute to the body of literature around their treatment.

In order to counsel patients, consideration must be given to the rupture risk of both the AVM and the prenidal aneurysm in these rare cases. Firstly, the presence of a prenidal aneurysm is an independent risk factor for AVM rupture and poor outcome. In fact, the PICA-aneurysm-AVM complex has an exceedingly high risk of rupture compared to other intracranial vessels (6). In addition, the aneurysm is the lesion that has a higher risk of bleeding and not the AVM (6). Such information is of considerable importance as far as treatment priorities are concerned.

## Case Report

A 68-year-old, right-handed male presented with episodes of nausea and vomiting over the last year. These episodes could be precipitated by sudden head movement, physical exertion, or sometimes in response to changes in ambient temperature. The severity and frequency of the episodes gradually progressed. Mild gait instability was also noted. His only other medical history included hypothyroidism and dyslipidemia. There was no family history of cerebral aneurysms. There was no history of smoking or excess alcohol consumption. On examination, he had difficulty performing tandem gait but had no other neurologic deficits. No other cerebellar signs were noted.

**Figure 1:**
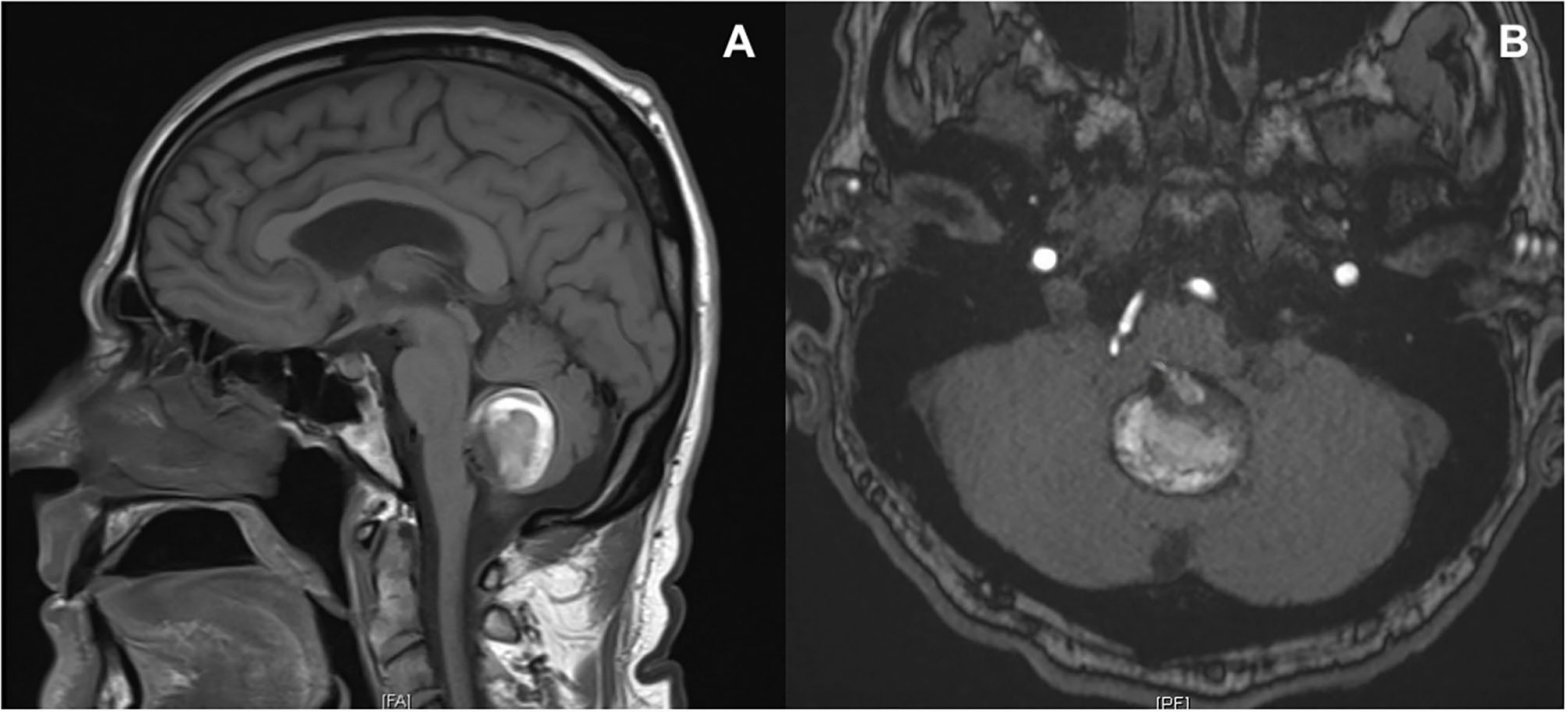
MRI T1 sequence **(A)** and time of flight acquisition **(B)**. Centered within the midline of the posterior fossa, favored to lie just below the floor of the fourth ventricle, there is a 3.1 × 3.1 × 2.8 cm partially thrombosed aneurysm sac, which arises from the right PICA. There is a suspected patent lumen measuring ~8.5 × 5.5 mm. Aneurysm sac demonstrates a narrow neck, ~2 mm. There is no evidence of superficial siderosis or hemosiderin deposition.

**Figure 2:**
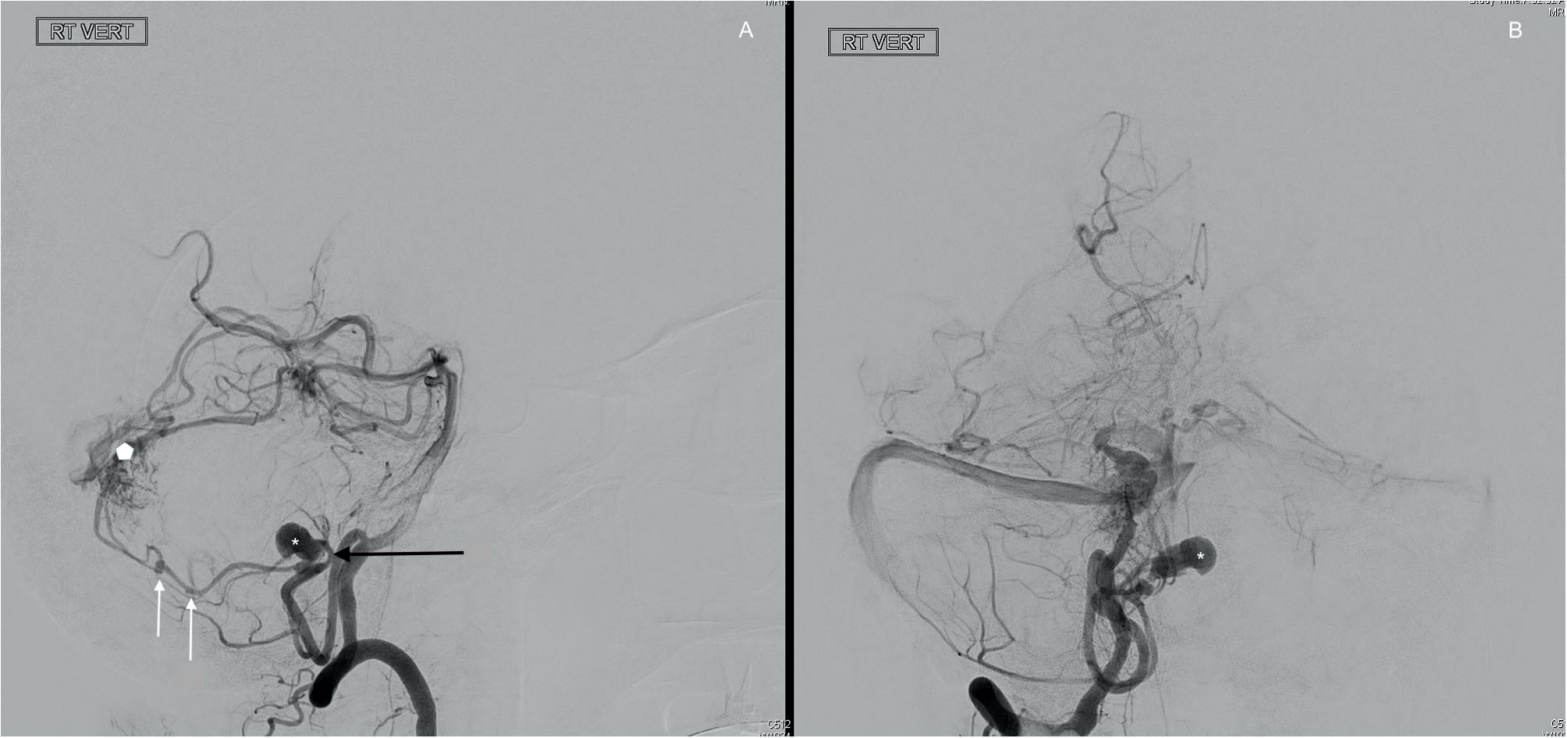
Digital subtraction angiography. Injection of the dominant right vertebral artery lateral **(A)** and AP view **(B)**. There is filling of the giant partially thrombosed aneurysm (asterisk), arising from a large right PICA distal to the caudal loop. Just proximal to the filling portion of the aneurysm there is a reduction in caliber of the PICA (black arrow on **A**). There appear to be 2 smaller aneurysms arising from the distal PICA (white arrows on **A**). The filling portion of the largest partially thrombosed aneurysm measures approximately 7.5 mm × 14 mm, the second aneurysm measuring approximately 4.0 x 2.6 mm and the most distal aneurysm measuring ~2.7 × 1.8 mm. A small arteriovenous malformation (white pentagon on A) is also seen with feeders from the right PICA and right SCA. Drainage of the AVM is via the torcula into the right transverse sinus **(B)**.

An MRI of the brain, arranged by his family physician, demonstrated a partially thrombosed giant right PICA aneurysm filling the fourth ventricle (Figure 1). The filling (non-thrombosed) component of the aneurysm measured 5 × 8 mm with a 2 mm neck. Mass effect was noted on bilateral dentate nuclei and cerebellar white matter as well as the pons. No AVM was detected on the MRI. There was no evidence of obstructive hydrocephalus. Conventional cerebral angiogram demonstrated that this aneurysm was arising from the right PICA on a segment distal to the caudal loop. It also showed a midline cerebellar AVM, with a nidus <3 cm, supplied by the right PICA. Another two smaller prenidal aneurysms were also noted more distally along the right PICA. In addition, the AVM is also supplied by the ipsilateral superior cerebellar artery (SCA) and drains superficially into the right transverse sinus through the torcula, Spetzler-Martin grade 1 (Figure 2). The Right vertebral artery was dominant in this case, and each PICA arises from the V4 segment of the respective ipsilateral vertebral artery.

After a detailed discussion with the patient and a review of both endovascular and open microsurgical options, the patient underwent endovascular treatment of the giant aneurysm with coil embolization/parent vessel occlusion (treatment decision and rationale are discussed below). Though all approaches, including endovascular and microsurgical interventions, have been applied to this specific lesion complex of multiple pedicle PICA aneurysm and AVM (7), the treatment of the AVM was deferred in this case. This is mainly due to the patient's age, the asymptomatic nature of the AVM, and the relatively lower risk of rupture of the AVM.

## Treatment

**Figure 3:**
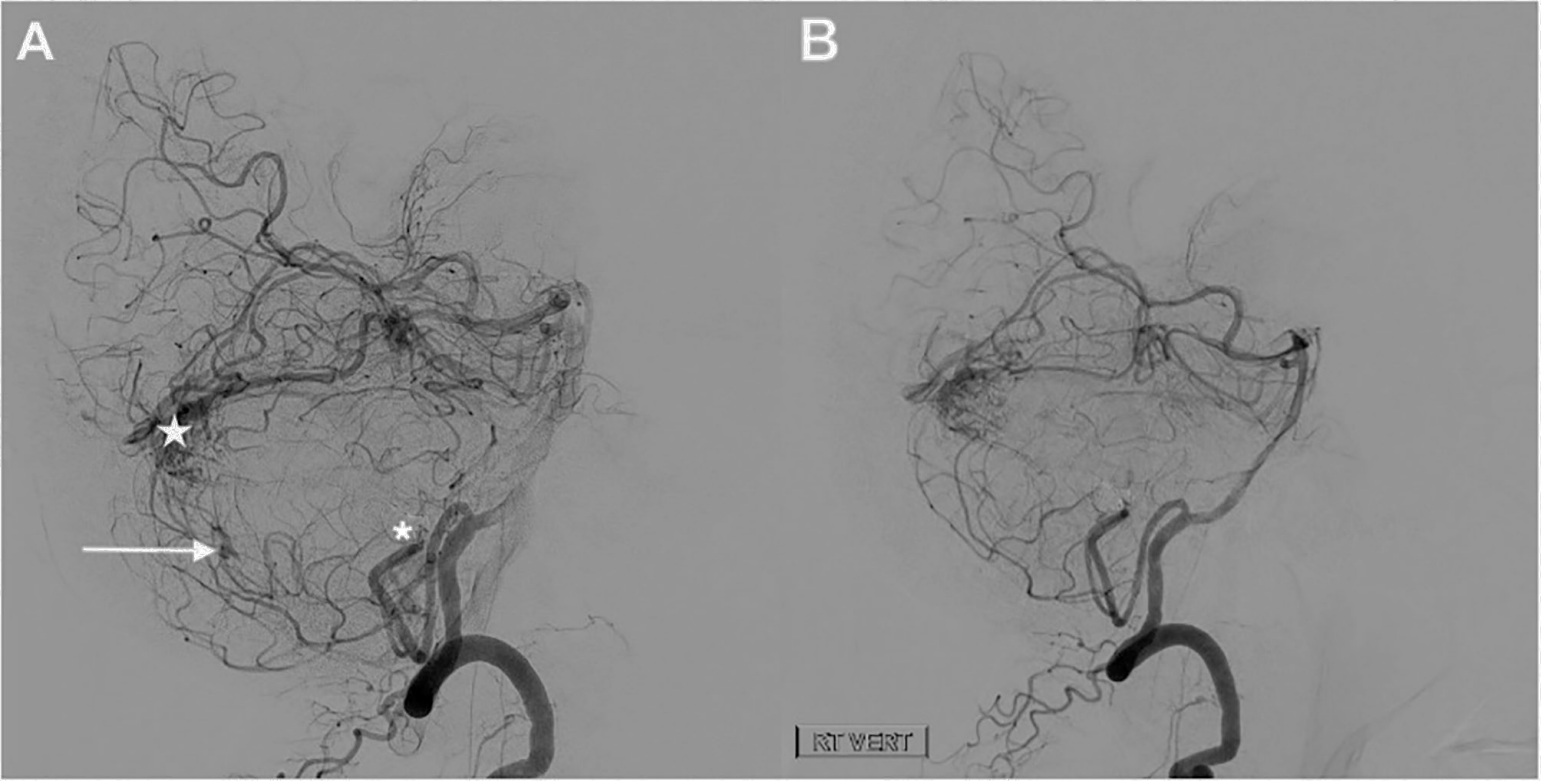
Digital subtraction angiography. Injection of the dominant right vertebral artery lateral view immediately after the embolization **(A)** and at 1 year later **(B)**. The two largest aneurysms were treated endovascularly via coil occlusion/embolization of the right PICA (parent vessel sacrifice). At the end of the procedure, there is no residual filling of the giant aneurysm (asterisks on **A**) or the second adjacent smaller aneurysm, but there was reconstitution of the distal PICA via collaterals with filling of the smallest distal aneurysm (white arrow on A) which was spontaneously obliterated at 1 year follow up **(B)**. There remains a small arteriovenous malformation (white star on **A**) fed by the right SCA and right PICA.

Under general anesthesia and after obtaining a femoral access, the aneurysm was approached using a 6 French guiding catheter (Envoy, Codman) and a micro catheter (Excelsior SL 10, Stryker). Using detachable coils, the distal segment of the PICA just proximal to the aneurysm was sacrificed including the aneurysm sac. Post coiling, angiography showed no residual filling of the aneurysm and occlusion of the right PICA at the inflow site to the giant aneurysm (Figure 3). There was distal reconstitution of the right PICA via collaterals at the level of the third aneurysm, which was proximal to the AVM. At this point, the procedure was stopped. The patient tolerated the procedure with no complications and was discharged home the following day. Angiography done a year later showed resolution of all aneurysms (Figure 3).

**Figure 4:**
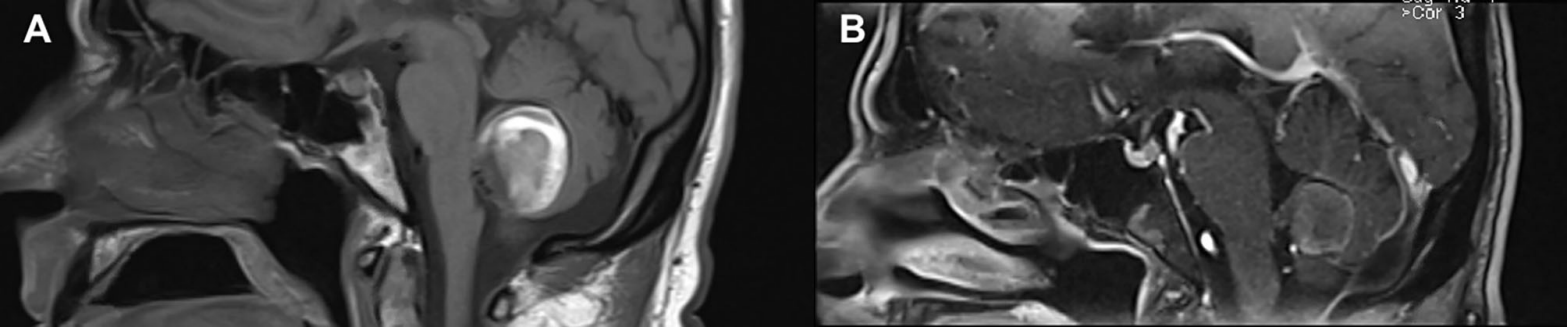
MRI comparison before treatment **(A)** and at 9 months follow up **(B)**. In follow up, there is no further filling of the giant aneurysm, and the thrombosed aneurysm sac measures 2.1 cm × 2.3 cm × 2.2 cm compared to 3.1 cm × 3.1 cm × 2.8 cm prior to treatment.

At the 9-month follow up, an MRI showed a reduction in the size of the thrombosed aneurysm, now measuring 2.1 cm × 2.2 cm × 2.3 cm, with no evidence of recanalization. The patient reported improvement of his nausea and vomiting as well as his gait with no new complaints reported (Figure 4).

## Discussion

### A Comparison With the Current Literature

The current paper reports the first case of a giant prenidal PICA aneurysm in the fourth ventricle, associated with a small midline cerebellar AVM. Our review of literature did not identify any similar cases of a giant PICA aneurysm in combination with an AVM at this particular location.

A literature search for PICA-AVM associated with distal aneurysm revealed a total of 57 cases previously reported with clear and adequate description. Most of these cases are reported within other larger series of distal PICA aneurysms (8). The prevalence of associated AVM ranged from 30 to 40% of all distal PICA aneurysms currently reported (5). From the reported cases, the average diameter of prenidal distal PICA aneurysms ranges from 4 to 6 mm with no giant aneurysm previously reported. In all reviewed cases with both PICA aneurysm and AVM, the aneurysm was consistently associated with higher risk of rupture. A detailed description of the previous series is provided (Appendix 1).

### Prenidal Aneurysm and Associated AVM: Pathophysiologic Relationship

The exact mechanism of how an AVM and prenidal/intranidal aneurysm develop is unknown. There are at least three different theories describing this relationship. The first (and least favored) was described by Boyd-Wilson in 1959 where this association was considered a random coincidence (3). This explanation fell out of favor due to convincing evidence that aneurysms associated with AVMs are much more common than the frequency of aneurysms in the general population (1). The second theory suggests that a common congenital defect is underlying the development of both the aneurysm and the AVM (4). However, no specific structural defect or genetic abnormality has been definitively identified as a cause for the development of both lesions. Lastly, the hyperdynamic theory provided by McKissock and Paterson in 1956 proposes that an aneurysm could develop in response to the hemodynamic stress imposed on the AVM-feeding vessels (4, 6). This theory is supported by the observation that treatment of the AVM can sometimes produce regression of an untreated prenidal aneurysm.

### Treatment Decision: Stepwise Management Approach

#### Surgical Clipping vs. Endovascular Coiling

PICA aneurysms are diverse in terms of location and morphology. No single treatment modality can therefore be consistently applied to all PICA aneurysms. A recent retrospective study reported at least nine different treatment modalities used for PICA aneurysms (including open microsurgical as well as endovascular methods) (9). To date, there is no head-to-head comparison between endovascular and open microsurgical approaches. The treatment of choice depends on aneurysm morphology, location, and surgeon/interventionist preference. When considering aneurysm location, PICA aneurysms can be classified as proximal (including those arising at origin of the PICA-vertebral junction or within the anteromedullary segment) and distal (located after the anteromedullary segment) (9). Most PICA aneurysms arise at the junction of the PICA and vertebral artery, and approximately one third arise more distally. Those that arise in distal segments are more often AVM related (prenidal); this is similar to the index case of this study (6). In addition, the proximal segment of the PICA courses in close relation to the brainstem and lower cranial nerves (XI-XI), which represents a microsurgical challenge. This segment also supplies a number of brainstem perforators, and occlusion of the PICA at this level would therefore likely result in brainstem ischemia. Together the PICA's variable origin, tortuous course, and close proximity, vital neurovascular structures pose challenges for both endovascular and open surgical approaches (8).

Having said this, the open surgical option was less favored for the case presented in this study. This is mainly due to the fact that the patient was initially intact with no neurological deficits and the high possibility of surgical-related morbidity. This includes injury to lower cranial nerves or brainstem perforators. In addition, the aneurysm and the AVM are located relatively far apart, which poses a significant surgical challenge to target both lesions with one approach. On the other hand, the endovascular option, in this situation, would provide a less invasive alternative that can target both lesions at once. In addition, there were a number of important characteristics that favor endovascular vessel occlusion present in this patient. These include the distal location of the aneurysm that spares most of the brainstem perforators as well as the favorable vascular morphology (see next section).

#### Endovascular Coiling With Parent Vessel Occlusion

The angioarchitecture of this particular aneurysm includes a complex morphology and a giant partially thrombosed dome that would make it unsuitable for clipping or primary coiling. Parent vessel occlusion would target both the aneurysm and the main arterial feeder to the AVM. Occluding the PICA in a distal location would also spare the brainstem perforators.

Reviewing the literature, parent vessel occlusion is considered an appealing option for treating distal PICA aneurysms; especially with dissecting pseudoaneurysms or those with complex morphology (9). For a dissecting pseudoaneurysm, parent vessel occlusion is recommended to secure the aneurysm and the proximal parent artery. The risk of causing an ischemic infarct secondary to endovascular treatment is low in the distal PICA territory compared to clipping (9). By reviewing the literature, ischemic complications have been reported to be low (8). In many cases, unilateral distal PICA occlusion will not cause infarction due to collaterals from other cerebellar arteries (5). In addition, since most brainstem perforators take off at the anterior and lateral medullary segment, parent vessel sacrifice distal to these two segments should spare these brainstem perforators. A recent study reported a center experience of parent vessel occlusion alone for distal PICA aneurysm and associated AVM with favorable outcomes (5).

#### Follow Up and Future Therapeutic Planning

At 9-month follow up, there was no further filling of the giant aneurysm and a significant reduction in mass effect. No brainstem ischemia or infarction were noted. Obliteration of the second and third aneurysms, located more along the course of PICA, was also noted, perhaps owing to the decrease in flow after parent vessel occlusion. The AVM, which itself has two patent feeders coming from the SCA, is still filling (Figure 3). The magnitude of risk associated with the residual AVM is unknown. There is a paucity of natural history data describing such partially treated AVMs. However, it has been reported that such an AVM can bleed after treatment of the prenidal aneurysm alone (6). Some authors suggest that microsurgical just microsurgical resection resection of the AVM with or without preoperative embolization is optimal in these cases; however, the locations of the aneurysm and AVM would determine whether this could be safely achievable (9). Since there is no consensus on the ideal treatment strategy, these cases should be assessed on an individual basis, taking into account the AVM size, complexity, location, and patient perspective. After a thorough discussion with the patient, the plan at this stage is to proceed with preoperative endovascular embolization of the AVM nidus via SCA feeding branches, using embolic material. This will be followed by microsurgical resection of the AVM via suboccipital approach. Thus, the planned intervention is to gain an intra-arterial approach using a femoral access, navigating through (RT SCA) using a guiding catheter and a microcatheter. Embolization is to be achieved using (detachable coils/embolic material-to be specified). Surgical resection through a midine suboccipital approach is the next option to consider if endovscular embilization fails to embolize the AVM.

## Conclusion

PICA aneurysms associated with an AVM are rare and clinically challenging entities. This paper presents the first case of a giant prenidal PICA aneurysm associated with a cerebellar AVM. Thorough evaluation of the pertinent anatomy, location, and morphology of both lesions, along with meticulous assessment of comparative risk of rupture, is of paramount importance. Parent artery occlusion can be a useful and well-tolerated option in treating distal PICA aneurysms with minor risk of ischemia. Involvement of a multidisciplinary team is a key for diligent planning of treatment, considering the complexity of these lesions. The information provided is this report is meant to assist in the decision-making process.

## Data Availability Statement

The original contributions presented in the study are included in the article/Supplementary Materials, further inquiries can be directed to the corresponding author.

## Ethics Statement

Ethical review and approval was not required for the study on human participants in accordance with the local legislation and institutional requirements. Written informed consent for participation was not required for this study in accordance with the national legislation and the institutional requirements.

## Author Contributions

HK: conceptualization, data curation, formal analysis, investigation, methodology, resources, visualization, writing—original draft, and writing—review and editing. CH: data curation, formal analysis, investigation, software, validation, visualization, and writing—review and editing. AA: conceptualization, project administration, supervision, validation, visualization, and writing—review and editing. All authors contributed to the article and approved the submitted version.

## Conflict of Interest

The authors declare that the research was conducted in the absence of any commercial or financial relationships that could be construed as a potential conflict of interest.

## Abbreviations

AVM: Arteriovenous malformation
PICA: Posterior inferior cerebellar artery
SAH: subarachnoid hemorrhage
SCA: Superior cerebellar artery.
