# Supplementary material for: Case Report: Endovascular Treatment of a Giant Distal PICA Aneurysm in Association With a Cerebellar AVM: A Report on Treatment Considerations and a Literature Review
Source: Front Neurol. 2020 Dec 18;11:611377. doi: 10.3389/fneur.2020.611377 (PMC7775600; doi:10.3389/fneur.2020.611377)
Supplement: Supplementary file 1 [file Table_1.DOCX]

Appendix 1: Summary of reported cases of distal PICA aneurysms and AVMs:

| Study | Total Patients | Total Aneurysms | Mean size of aneurysm(mm) | Associated Avm* | Treatment for prenidal aneurysm (number)** | Treatment for AVM | Ruptured aneurysm (% of all aneurysms) | Ruptured AVMs (% of all AVMs) |
| --- | --- | --- | --- | --- | --- | --- | --- | --- |
| Hudgins 1983 (1) | 4 | 4 | - | 2 | - | - | - | - |
| Gacs 1983 (2) | 8 | 8 | - | 2 | - | - | - | - |
| Bertalanffy et al., 1998 (3) | 9 | 9 | - | 3 | Clip (3) | Resection (3) | - | - |
| Captain et al 1999 (4) | 27 | 27 | 5.5 | 27 | Trap (8)  Clip (13)  None (6) | Resection (27) | 25 (92) | 2(7.4) |
| Bradac et al., 2004 (5) | 6 | 6 | - | 1 | PVO (1) | None (1) | 6 (100) | 0 |
| Bohnstedt et al., 201511(6) | 23 | 23 | 7 | 2 | Coiling (2) | None (1) | 19 (82) | 0 |
| Lewis et al., 2002 (7) | 20 | 22 | 6 | 6 | Clip (2)  PVO (4) | Resection (6) | - | - |
| Lheto 2014 (8) | 58 | 91 | 10.5 | 4 | Clip (2) | Resection (2)  PVO (1)  N/A (1) | 68 (75) | 1 (25) |
| Li et al., 2008 (9) | 5 | 6 | - | 1 | None (1) | None (1) | - | - |
| Orakcioglu et al., 2005 (10) | 16 | 18 | 5 | 1 | Clip (1) | Resection (1) | 15 (93) | 0 |
| Williamson et al., 2015 (11) | 22 | 22 | 6 | 1 | Clip (1) | Resection (1) | 22 (100) | 0 |
| Zhou et al., 2010 (12) | 6 | 6 | 6.2 | 1 | PVO (1) | Resection (1) | 6 (100) | 0 |
| Case et al., 2016 (13) | 12 | 14 | 5 | 5 | PVO (5) | Embolization (3)  deferred (1)  Gamma knife (1) | 9 (75) | 0 |
| Nonaka et al., 2018 (14) | 1 | 2 | - | 1 | Trap (1) | Resection (1) | - | - |

*: Number of patients diagnosed with AVM in association with distal PICA aneurysm; the aneurysm is pedicular in all cases and located on PICA that is feeding the AVM.

**: shown here is treatment strategy only for aneurysm found in association with AVM; not all other aneurysms in the paper.

PVO: parent vessel occlusion; N/A: not available

Refrences:

1. Hudgins RJ, Day Al, Quisling RG, Rhoton AL, Sypert GW, Garcia-Bengochea F. Aneurysms of the posterior inferior cerebellar artery. A clinical and anatomic analysis. J Neurosurg. 1983;58: 381-387.
2. Gács G, Viñuela F, Fox AJ, Drake CG. Peripheral aneurysms of the cerebellar arteries. Review of 16 cases. J Neurosurg. 1983;58:63-68.
3. Bertalanffy H, Sure U, Petermeyer M, Becker R, Gilsbach JM. Management of aneurysms of the vertebral artery-posterior inferior cerebellar artery complex. Neurol Med Chir (Tokyo). 1998;38(suppl):93-103.
4. Kaptain GJ, Lanzino G, Do HM, Kassell NF. Posterior inferior cerebellar artery aneurysms associated with posterior fossa arteriovenous malformation: report of five cases and literature review. Surg Neurol. 1999;51:146-152.
5. Bradac GB, Bergui M. Endovascular treatment of the posterior inferior cerebellar artery aneurysms. Neuroradiology. 2004;46:1006-1011.
6. Bohnstedt B, Ziemba-Davis M, Edwards G, Brom J, Payner T, Leipzig J, et al. Treatment and outcomes among 102 posterior inferior cerebellar artery aneurysms: a comparison of endovascular and microsurgical clip ligation. World Neurosurg. 2015;83:784-793.
7. Lewis SB, Chang DJ, Peace DA, Lafrentz PJ, Day AL. Distal posterior inferior cerebellar artery aneurysms: clinical features and management. J Neurosurg. 2002;97:756-766.
8. Lheto H, Harati A, Niemelä M, Dashti R, Laakso A, Elsharkawy A, et al. Distal posterior inferior cerebellar artery aneurysms: clinical features and outcome of 80 patients. World Neurosurg. 2014;82:702-713.
9. Li XE, Wang YY, Li G, Jia DZ, Liu XH, Gao J, et al. Clinical presentation and treatment of distal posterior inferior cerebellar artery aneurysms: report on 5 cases. Surg Neurol. 2008;70:425-430.
10. Orakcioglu B, Schuknecht B, Otani N, Khan N, Imhof HG, Yonekawa Y. Distal posterior inferior cerebellar artery aneurysms: clinical characteristics and surgical management. Acta Neurochir (Wien). 2005;147:1131-1139.
11. Williamson R, Wilson D, Abla A, McDougall C, Nakaji P, Albuquerque F, et al. Clinical characteristics and long-term outcomes in patients with ruptured posterior inferior cerebellar artery aneurysms: a comparative analysis. J Neurosurg. 2015; 123:441-445.
12. Zhou Y, Kato Y, Olugbenga O, Hirotoshi S, Karagiozov K, Masahiro O, et al. The true distal posterior inferior cerebellar artery aneurysm: clinical characteristics and strategy for treatment. Minim Invasive Neurosurg. 2010;53:9-14.
13. Case, David, David Kumpe, Luis Cava, Robert Neumann, Andrew White, Christopher Roark, and Joshua Seinfeld. “Ruptured Distal Posterior Inferior Cerebellar Artery (PICA) Aneurysms Associated with Cerebellar Arterial Venous Malformations (AVMs): A Case Series and Review of the Literature Demonstrating the Need for Angiographic Evaluation and Feasibility of Endovascular Treatment.” World Neurosurgery 97 (2017). <https://doi.org/10.1016/j.wneu.2016.10.081>
14. Nonaka, Senshu, Hidenori Oishi, Satoshi Tsutsumi, and Hisato Ishii. “Posterior Fossa Arteriovenous Malformation with Multiple Pedicle Aneurysms and Recruitment of Meningeal Supply.” Asian Journal of Neurosurgery 13, no. 4 (2018): 1250. <https://doi.org/10.4103/ajns.ajns_180_17>
